# Depression, Constraint, and the Liver: (Dis)assembling the Treatment of Emotion-Related Disorders in Chinese Medicine

**DOI:** 10.1007/s11013-012-9290-y

**Published:** 2013-01-12

**Authors:** Volker Scheid

**Affiliations:** EASTmedicine Research Centre, School of Life Sciences, University of Westminster, 115 New Cavendish Street, London, W1W 6UW UK

**Keywords:** China, Depression, Emotion-related disorders, Cross-cultural psychiatry, Chinese medicine

## Abstract

Traditional Chinese medicine (TCM) is today practiced worldwide, rivaling biomedicine in terms of its globalization. One of the most common TCM diagnoses is “Liver qi constraint,” which, in turn, is commonly treated by an herbal formula dating back to the 10th century. In everyday TCM practice, biomedical disease categories such as depression or anxiety and popular disease categories such as stress are often conflated with the Chinese medical notion of constraint. Medical anthropologists, meanwhile, argue that constraint reveals to us a distinctive aesthetics of constructing body/persons in Chinese culture, while psychologists seek to define constraint as a distinctive psychiatric disorder distinctive from depression and anxiety. All of these actors agree in defining constraint as a concept dating back two thousand years to the very origins of Chinese medicine. This article disassembles the articulations by means of which these different facts about constraint are constructed. It shows how ideas about constraint as a disorder caused by the penetration of external pathogens into the body were gradually transformed from the eleventh century onward into constraint as an emotion-related disorder, while treatment strategies were adjusted to match perceptions about body/self that developed among the gentry elite of southeast China in late imperial China.

## Introduction

The essential characteristic of a nation is that all its individuals must have many things in common … And must have forgotten many things as well. (Ernest Renan)

The cover page of *Transforming Emotions with Chinese Medicine* by Yanhua Zhang (2007), a wonderfully perceptive ethnography of the sensibilities that inform Chinese medicine doctors in Beijing in their treatment of emotion-related disorders,^1^ shows a single character written in traditional Chinese—鬱 (*yù*). Variously rendered in the English language literature on Chinese medicine as “stagnation,” “depression,” “blockage,” or “constraint” (the translation that I favor and will use in the remainder of this article), the character’s contemporary meanings allude not only to dense luxuriant foliage, lush verdant growth, fragrance, and elegance but also to gloomy, depressed, and dejected moods, or pent-up frustration. Its original definitions include that of a thicket that hinders movement and of a tone that does not carry, readily explaining its apparently opposite connotations (Luo Zhufen 羅竹風 1997, p. 1924). To Chinese speakers today constraint thus can refer to both the emotional and physical feelings of blockage that are a common symptom of emotion-related disorders. Trained Chinese medical doctors discover constraint not only by asking and observing but also by palpating the pulse or the abdomen. In all of these senses the notion of constraint readily cuts through the psyche/soma dichotomies that inform modern western conceptions of mental illness. Zhang, therefore, interprets constraint and the practices into which it is embedded as denoting a uniquely Chinese aesthetic of fashioning the body/person.

Siu-man Ng and his collaborators at the University of Hong Kong come to very different conclusions. They pull the notion of constraint away from concerns with culturally specific practices and ways of being in the world toward a universally shared experience of being ill. Based on data collected in Hong Kong Ng’s research team has constructed an instrument that validates constraint as a unique psychiatric disorder related to but distinctively different from anxiety and depression. The group currently seeks to establish international collaborations that will test “the robustness of the new concept in different cultures” and “facilitate the sharing of health wisdoms from different parts of the world” (Ng et al. 2006, 2011).

No one seems to have taken up Ng’s invitation to date. The international biomedical community, as Suh (this issue) shows for the related case of *hwa*-*byung* in Korea, is interested in psychological disorders defined in Asia by Asians only if they are packaged as culture-bound syndromes or stripped of anything that might tie them to their specific points of origin. Yet, unlike *hwa*-*byung* and via quite different routes than those imagined by Ng, constraint has already succeeded in going global. It is one of the most common diagnoses in the contemporary practice of traditional Chinese medicine (TCM), and as TCM has become a global phenomenon, so, too, has constraint. To be more precise, what has gone global is not some ancient Chinese notion of experiencing the self, but a quite modern understanding of the disease process that tends to define constraint as a pathology associated most closely with the Liver organ system (肝 *gān*) in Chinese medicine on the one hand and with contemporary notions of stress, depression, emotion, and self on the other.^2^

I first encountered this condition as a TCM student in the UK in the early 1980s. Patients who complained of feeling stressed, irritable, or frustrated invariably were diagnosed as suffering from “Liver *qi* constraint.” This included premenstrual women, middle-aged managers, angry teenagers, and pensioners. Different types of body pain, bloating, and physical discomfort, virtually any symptom with a tendency to come and go, and specifically those symptoms that were associated with some kind of emotional trigger indicated that Liver *qi* was stuck and that this blockage had to be resolved with acupuncture or herbal medicines—sometimes successfully and sometimes not.

These personal observations are by no means exceptional. A popular acupuncture referral website in the United States contains a page dedicated to “The Liver and Liver Qi Stagnation” (Joswick 2010). Its author explains that “Liver *qi* stagnation, is one of the most common imbalances treated by Eastern medicine practitioners in the United States” because it corresponds to conditions caused by stress and emotional problems. Halfway around the globe, the Chinese medicine blogger Liang Jinghui 梁景輝 (2010) observes a similar prevalence of the condition in Taiwan and for the very same reasons. Yanhua Zhang (2007, pp. 85–87) states that during her own fieldwork in Beijing she, too, quickly started to associate emotion-related disorders quite narrowly with Liver *qi* constraint. Modern TCM textbooks, as Karchmer (this issue) confirms, tend to define constraint as an emotion-related disorder (or vice versa) and primarily link both to dysfunctions of the Liver organ system (Deng Tietao 鄧鐵濤 1987; Qiao Mingqi 喬明琦 and Zhang Minyun 張惠雲 2009, p. 119; Tan Kaiqing 譚開清 1998; Zhang Baiyu 張伯輿 1988; Zhao Guoyang 趙國祥 2009).

Around the world, the most frequently prescribed remedy for Liver *qi* constraint today is a herbal formula known as *xiāo yáo sǎn* 逍遙散, commonly translated into English as *Rambling Powder* (Scheid et al. 2009, pp. 120–125).^3^ *Rambling Powder* is one of five formulas suggested for the treatment of constraint disorders by the semiofficial *Chinese Medicine Protocols for Treating Diseases Based on Patterns* 中醫病證治療常規 (Zhang Ruhong 章如虹 et al. 1997). In 2009/10 alone, one of the UK’s leading suppliers of Chinese herbal medicines sold approximately 80,000 bottles of *Rambling Powder* pills. This represents 45 % of the company’s turnover of patent medicines during that year (Plant 2011). Similar figures were supplied to me by another leading supplier in the UK (Chen 2011), and confirmed by conversations with pharmacists in the US (Castle 2011).^4^ Data from a Chinese medicine teaching clinic in the UK shows that 27.6 % of all prescriptions written over the course of 1 year were modifications of *Rambling Powder*, with a diagnosis of Liver *qi* constraint the most typical indication (Mcgechie 2009). Mediated by diagnoses of Liver *qi* constraint *Rambling Powder* is also the most commonly used formula in clinical studies that seek to evaluate the effectiveness of Chinese medicine for emotion-related disorders, specifically for depression (Butler and Pilkington 2011).^5^ Hazy linguistic boundaries (in Chinese) between the Chinese medical concept of “constraint” 鬱証 (*yùzhèng*) and the biomedical diagnosis of “depression” 抑鬱症 (*yìyùzhèng*) are thereby conveniently replicated at the bedside and thence translated into clinical studies.

These observations allow a glimpse of the kind of complex articulations through which constraint has entered into the life-worlds of people suffering from tension, stress, and other emotion-related disorders around the world. Pace Zhang and Ng, these articulations do not depend on either patients or physicians being Chinese, or require validation by ICD, DSM, and the international psychiatric community. Nor do they respect established cultural systems of meaning. In Chinese medicine constraint is a pathology of the movement and diffusion of energy and matter, of *qi* 氣, blood (血 *xuè*), and body fluids (津液 *jīnyè*). Yanhua Zhang (2007, p. 45) argues that such constraint denotes a blockage of flow that is categorically different from the notion of depression, a concept universally associated with “soul loss” and feelings of emptiness. Nevertheless, TCM practitioners throughout the world today equate stress with Liver *qi* constraint and translate diagnoses of depression into processes of stagnation. Though beyond the creation of such equivalences they are rarely able to agree on very much else: neither on what actually is constrained, nor on what causes this constraint, and certainly not on how to treat it.

This paper, like the others collected in this special edition, critiques the simplifications and essentialisms that permeate current discourses on constraint within and outside of Chinese and other East Asian medicines. Besides a striving for historical accuracy and analytical rigor we also wish to contribute on a very practical level to the framing of questions in clinical research. Before spending potentially large sums of money evaluating whether or not a given Chinese medicine formula or treatment can treat depression, for instance, it would be wise to ask at least the following questions: What makes the diagnosis of Liver *qi* stagnation such an obvious match for a biomedically defined disorder that few people in China knew before the 1980s? What among the thousands of formulas in the archive of Chinese medicine singles out *Rambling Powder* to be the most obvious choice for treating depression, a choice promoted by official TCM institutions but not necessarily shared by Chinese, Korean, or Japanese physicians in their own clinical practices? This immediately leads to many other questions: about how *Rambling Powder* came to be connected to depression; about the articulation between East Asian medicines and modern psychiatry; and about the history of constraint itself.

More ambitiously, we believe that the notion of constraint and its diverse clinical uses afford a convenient springboard for asking wider questions about the relation of body/mind/emotion in health and disease and about the interface between apparently universal biologies and local embodiment. I say “apparently” because the globalization of constraint reminds us, and such reminders are still necessary, that “the universal” does not automatically equate with “the western” or “the biomedical.” Constraint, as Yanhua Zhang (2007) has demonstrated, originates in an enduringly Chinese concern for and attention to movement and flow. Yet, as these concerns were passed down across time and space, first across East Asia and then the rest of the world, they had to be realized in ever-changing contexts of practice that forced redefinitions of what constraint meant to patients and physicians and thereby also their own experiences of themselves. It is these changes over time and the consequences thereof that we wish to explore.

If a model is needed to guide such inquiry, few can be more appropriate than Ludwik Fleck’s (1980) seminal essay *The* *Genesis and Development of a Scientific Fact*. Using the discovery of the Wasserman reaction as a diagnostic indicator for syphilis as a case study, Fleck argued that facts emerge at the intersection of the interrelated pathways that the title of his essay alluded to. Genesis, the first of these, attends to the diachronic trajectory of an event horizon that establishes what practices become possible at any given moment in time. Fleck examines this trajectory for the case of syphilis by outlining a four-stage development of the underlying disease concept through which it was defined: from a carnal scourge implying sin, to one of befouled blood, to the concept of a curable condition, and finally to the identification of a causative agent (Hedfors 2006).^6^ Development, the second trajectory, examines the processes of articulation that transforms potentiality into what becomes real in the here-and-now, and that in doing so vanquish alternative possibilities—at least for the present time. In Fleck’s example, this development is traced in the coming together of the Wasserman reaction through the action of numerous interrelated agents, many of them non-scientists, all with their own agendas.

In that sense and for the reasons outlined above, my investigation will seek to disassemble the TCM practices that articulate the notion of constraint with the Liver organ system, *Rambling Powder*, and both folk and professional discourses on mental health in China and the West. Focusing on China and covering the rather long period from the 4th century BCE to the early 19th century CE, it is weighted toward the diachronic or genesis pole of Fleck’s dichotomy. In doing so, it allows the other three papers collected in this issue to focus on the development of specific types of knowledge and practice at distinctive moments in time.

To this end I will outline the history of constraint in imperial China as a gradual coming together of a number of originally unconnected aspects of doctrine and practice. These include: first, a shift in the definition of constraint from a disorder caused by external disease causes to one involving the emotions; second, a transformation in the conception of constraint from one centered on the Lungs to one that primarily involved the Liver and that, in turn, was related to a shift in emphasis from the pathophysiology of body fluids to one that focused on physiological fire; third, the un-gendering of constraint from a disorder mainly of women to one that also afflicted men; and fourth, the reconceptualization of constraint as a deficiency disorder. By the early 19th century, these different ideas had been assembled into a distinctive and widely shared style of practice that dominated Chinese medical practice in southeast China but not necessarily elsewhere. This practice articulated constraint with emotion-related disorders, with the Liver as the primary site of physiological disturbance, and suggested *Rambling Powder* as a suitable treatment protocol. It was this assemblage that allowed physicians in 20th century China to link the indigenous discourse on constraint to ideas about nervous system disorders imported from the West as described by Karchmer. The papers by Daidoji and Suh focusing on Japan and Korea, meanwhile, alert us to the ever-present possibility of different kinds of assemblages that can be constructed from the same original ideas.

I will proceed chronologically beginning with the definition of constraint as a disease concept in pre-Han China, and then discuss key texts and physicians that shaped the trajectories of transformations alluded to above. I will establish that while there is linearity to such genesis, this does not imply a history of progress toward some necessary and predetermined endpoint, nor the simple re-enactment in different contexts of practice of some unchanging concepts, values, or orientations. Rather, as I have argued above, there are always choices to be made; and if there is any real value in an examination such as this, then it surely lies in understanding the past so as to facilitate the taking of decisions that have to be made in the present.

## The Origins of Constraint in East Asian Medicines

In an effort to put clinical practice on a more secure empirical basis, a group of revisionist physicians in 18th century Japan embarked on an ambitious project of medical reform that questioned each and every aspect of traditional practice. In spite of their generally critical attitude toward metaphysical speculation, the one single concept they never contemplated to abandon was that of *qi*. On the contrary, they elevated *qi* stagnation (滯 *zhì*) and constraint (鬱 *yù*) to the sole cause of all disease. Yoshimasu Tōdō 吉益東洞 (1702–1773), a leading representative of this movement, pointed to a passage from *The Annals of Lü Buwei* 呂氏春秋, an almanac of life in pre-Han China compiled around 239 BCE, to emphasize the long historical provenance of their revolutionary ideas (1747):

Flowing waters do not stagnate and door hinges do not get mole crickets. This is because they move. It is the same with respect to the bodily frame and *qi*. If the bodily frame does not move, the vital essences do not flow and the *qi* constrains. (Lü et al. 2000, p. 100)

Yoshimasu Tōdō’s strategy of anchoring his critique of received knowledge in even older textual sources, even as it emphasizes *qi* movement and flow as one of the central concerns of East Asian medicine, should not diminish the radical nature of his ideas. The authority that he rejected, after all, was that of the *Inner Canon of Huang Di* 黃帝內經 (comp. 4th–2nd century BCE), the foundational text of scholarly medicine in China whose status and influence throughout East Asia has been compared by medical historians to that of the *Corpus Hippocraticum* in the West (Unschuld et al. 2011, vol. 1, p. 11).

What the authors of the *Inner Canon* themselves had achieved—and what was later so forcefully criticized by Yoshimasu Tōdō in Japan as well as by his followers in Republican China—was the creation of a science of healthcare founded on models of systematic correspondence borrowed from natural philosophy. For instance, in order to treat blockages of physiological function, the authors of the *Inner Canon* drew on existing notions of constraint outlined above but re-classified them into five different types so as to align them with the wider organization of phenomena in the world based on the concept of the five phases (五行 *wǔ xíng*) (Unschuld et al. 2011, vol. 2, p. 531). Recommended treatment strategies for removing these blockages included emesis, diuresis, purging, and sweating, suggesting that the stagnations of *qi* flow discussed here were imagined as substantive obstructions that somehow needed to be eliminated from the body (See Table 1).

**Table 1:** The Five Types of Constraint in *Inner Canon: Basic Questions, chapter 71*

Constraint of	Wood *Qi*	Fire *Qi*	Earth *Qi*	Metal *Qi*	Water *Qi*
Pathology	Wind (obstruction of upward movement of *qi*)	Obstruction of yang *qi* (often by cold)	Obstruction of digestive function	Blockage of *qi* (downward movement)	*qi* counterflow (excessive upward movement)
Treatment	Emesis	Sweating, effusion	Purging, vomiting, draining	Stimulating urination	Curbing *qi* counterflow; opening the passage of water

In another passage of text, constraint is more specifically linked to the Lungs, whose control of breathing resonated with the rhythmic contraction and expansion imagined to be characteristic of all *qi* movement and transformation (Unschuld et al. 2011, vol. 2, p. 627). This articulation between constraint and the Lung’s regulation of *qi* would come to play an important aspect in the development of pharmacological treatments for constraint a thousand years later during the Song dynasty (960–1279).

To understand these later developments it is important to note that to the authors of the *Inner Canon* *qi* movement and transformation encompassed every aspect of human existence, including the flow and expression of emotion (Unschuld 2003, p. 228). Linked into the *qi* physiology that animated the human body/person at large, emotions could thus affect wellbeing in the very same way as environmental factors like cold, heat, or wind.^7^ How to channel the emotions in order to ensure a harmonious relationship between individual body/persons and their environment accordingly became a legitimate concern for the naturalist physicians that compiled the *Inner Canon*, opening up an entire field of practice to new medical interventions. However, none of the diverse voices within this young tradition had yet conceptualized constraint as a specific disease, emphasized its link to emotional disorders, or discussed its relation to the Liver organ system. The first step of the long process that would eventually effect these articulations involved the movement of constraint to the very heart of medical practice. This was carried out between the 12th and 14th centuries by a small group of scholar physicians in southern China, in response to state interventions into medical care and the emergence of Neoconfucianism as a new way to fashion the self.

## Making Constraint Central

Historians of medicine have not yet examined the specific reasons that turned constraint into a central concern for elite physicians in China from the 12th century onward. It is likely, however, there is at least some overlap with what happened several centuries later in Japan. Kuriyama (1997) has documented how the rapid commercialization of Japanese society during the Edo period (1615–1868), dependent on the circulation of money and goods in the world outside, was mirrored in increasing concerns about problems of stagnation and blockage within the human body; and how this matched the emergence of similar anxieties during the industrial revolution in the West. Daidoji (this issue) shows that Japanese physicians at the time blamed problems of *qi* constraint on idleness, affluence, and declining opportunities for venting frustration through more outright physical aggression.

Beginning in the Song dynasty, China underwent a process of increasing urbanization, the development of a cash based economy, and technological advances in many fields that resemble some of the later transformation in Japan and the West examined by Kuriyama and Daidoji. Feudal elites were replaced by a new gentry class whose members owed their status to positions in the state bureaucracy awarded after passing onerous civil service examinations. This caused students, scholars and their families to experience new stresses that played a part in the emergence of constraint as an important medical problem. At most times, the number of available positions far exceeded that of candidates with the required qualifications, and the number of students studying for the exams that of those who succeeded. As a result, frustration about the lack of opportunity for putting one’s talents to use and for social advancement more generally became endemic (Mote 1999). At the same time, male gender identities also underwent important transformations.

Spurned by the pursuit of examination success male members of the gentry elite increasingly valorized learning over martial skills and military pursuits. This tendency was exacerbated after the Mongol conquest of China in 1279, which made a bureaucratic career or the retreat into scholarly learning and teaching the only choice for these gentlemen. Gradually, over the course of subsequent centuries, elite males thus came to emphasize artistic sensibility, physical fragility, sexual passions, and even self-indulgence over physical strength and prowess, without thereby surrendering social dominance over women (Song 2004). Perceiving themselves as physically weak and consistently frustrated on many levels, these men had a tendency to become “hypochondriacs, obsessed with diets, medicines and health generally” (Elvin 1989, p. 267). Commenting on the life of the famous Song dynasty literary genius Su Shi 蘇軾 (1037–1101) one biographer notes that “with his inquiring mind, his (bumbling!) attempts to acquire an inner or outer elixir, and his sporadic ‘courses’ in breathing and meditation, [he] lived ahead of his time since he seems to have been the prototype, in more ways than one, of the typical twentieth-century neurotic” (Baldrian-Hussein 1996, p. 46). I prefer a less Orientalist reading that sees Su and his peers not living ahead of their time but in their time; not as neurotics diagnosed according to by now outdated biomedical disease categories, but as actively searching for ways to manage the predicaments of their lives. At times, they would enlist physicians in their personal quest for health and wellbeing and thereby contribute to changes in medical practice.

**Fig. 1:**
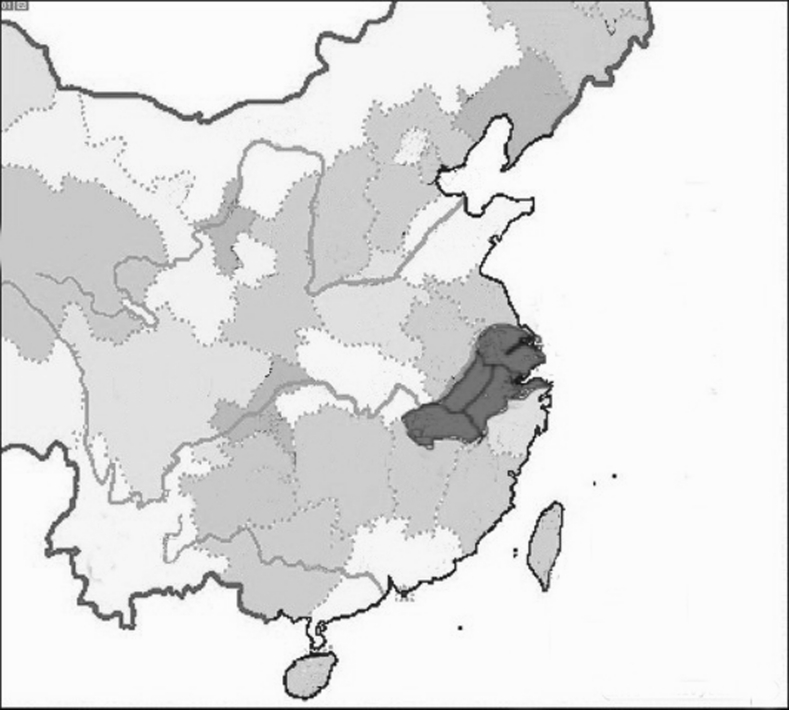
Map of Jiangnan

All of these developments were particularly pronounced in China’s southeast. Invading armies like the Jurchen, the Mongols, and later the Manchu invariably came from the north. Conversely, with the move of the capital to Hangzhou in 1127, the empire’s economic and cultural center shifted south. Over time, therefore, new modes of self-fashioning among the elite merged with regional identities to produce cultural stereotypes that viewed southerners as constitutionally weak and southern men, in particular, as feminized (Song 2004). Medical discourse naturally responded to, reproduced and amplified these stereotypes, specifically among the newly emergent scholar physicians who belonged to the same social strata as their gentry patients, spoke their language, and shared their concerns (Hanson 1998).^8^ In fact, all of the Chinese physicians whose contribution to the genesis of constraint I discuss in this essay come from the region of southeast China known as *Jiāngnán* 江南, which comprises what are today northern Zhejiang, southern Jiangsu, and eastern Anhui provinces (Fig. 1).

Among this group of doctors, the works of Chen Yan 陳言 (1131–1189) and Zhu Danxi 朱丹溪 (1281–1358) stand out for their influence on establishing constraint as a central concern of medical practice. Both doctors hailed from Zhejiang and drew on the same corpus of textual sources and medical technologies. Though they were both exemplars of elite medicine before and after it was swept up by the Neoconfucian transformation of Chinese thought, they differed considerably in how they brought these resources to bear on clinical practice.

## Chen Yan 陳言 (1131–1189)

Chen Yan was an old-style Confucian teacher who aimed to produce systematic, reliable, and effective medical knowledge by smoothing out differences between the multiple voices of tradition that had accumulated in his time. To this end, he sorted all illnesses into three groups according to their causes: external causes consisting of climatic *qi* that penetrated into the body from the outside; internal or emotional causes; and miscellaneous causes comprised by accidents, wounds, parasites, and other factors that would not fit into the primary external/internal dichotomy. In defining these causes Chen Yan explicitly associated constraint with emotion-related disorders:

The seven emotions constitute a person’s normal nature. When they are stirred they initially emit from their constraint in the organ systems, which [subsequently] takes on external form in the limbs and trunk. These are internal causes … (Chen Yan 陳言 1173, p. 36).

Careful examination of the disorders Chen Yan discusses under the heading of internal causes shows a further subdivision into two main types. The first of these comprises illnesses where disordered emotions manifest a dysfunction in one or more of the main organs systems. Excess Liver heat (肝實熱 *gān shí rè*), for instance, is said to manifest with pain in the flanks, flushing, wheezing, eye pain, and unclear vision, as well as with feelings of resentment, grief, anger, mania, and uncensored speech. Only for the second type of illnesses do emotions themselves constitute the main cause. Irrespective of the specific emotion involved emotional excess in these cases is imagined as blocking the movement of *qi*. This causes the secondary changes in fluid physiology and potentially even more serious accumulations in the body down the line:

When the *qi* of the organ systems stops moving, it constrains and produces drool. Following the flow of *qi* it gathers and accumulates, hardening it becomes big like a lump and is located between the Heart and the abdomen. Or, it obstructs the throat like a piece of cotton that can neither be coughed up nor swallowed. [These symptoms] come and go but each time one feels as if one wants to die. These manifestations are as if produced by spirits and lead to rejection of food and drink (Chen Yan 陳言 1173, p. 101).

Chen Yan thereby presents a systematic exposition of emotion-related disorders that clearly links causes, pathophysiological processes, and external manifestations along both somato-psychic and psycho-somatic illness pathways. Just as an aside, we may note that in doing so he challenges the simplistic opposition between modern/western psychosomatics and ancient/Chinese somatopsychics that dominate historical and ethnographic writings on the subject.^9^ More pertinent to Chen’s own time were the treatment strategies he outlined. These articulated the body of the *Inner Canon*, which, as we have seen, was centered on organ systems and processes of transformation understood by way of their integration into five phases metaphysics. Pharmacotherapy, on the other hand, was borrowed from the *Treatise on Cold Damage and Miscellaneous Disorders* 傷寒糴病論.^10^ Composed by Zhang Ji 張機 (150–219) toward the end of the Han dynasty, this text gained considerable importance from the 10th century onward following official endorsements by the Song state. The Song state also promoted pharmacotherapy more generally by establishing an Imperial Pharmacy 和劑局 in 1076. Over the next century, the Imperial Pharmacy compiled several official formularies and initiated the mass production and distribution of standardized prescriptions listed within these formularies (Goldschmidt 2009). Both of these initiatives influenced Chen Yan in his search for pharmacotherpeutic responses to emotionally caused constraint disorders.

For this he turned to *Treatise on Cold Damage and Miscellaneous Disorders*, which not only contains descriptions of several disorders that are clearly emotion-related but also lists herbal formulas for their treatment. The language in which these disorders are discussed points to roots in even older accounts of spirit possession, to which women were seen to be particularly prone. Chen Yan borrowed two of these formulas to treat his new category of emotionally caused disease but dropped their gender bias, effectively assimilating Cold Damage therapeutics to the un-gendered body of the *Inner Canon* (Furth 1999), which itself did not contain much in the way of pharmacotherapy. Yet, if Chen also continues to talk of diseases of the seven emotions “as if produced by spirits,” it suggests that naturalist explanations continued to compete with spirit-possession models even a thousand years after the compilation of his source texts.

Chen Yan did not elaborate on why his formulas might be effective. However, by linking the action of their main ingredients to his pathophysiological explanations, it is possible to reconstruct some of the reasoning underlying his thinking.^11^ Such an analysis strongly suggests that Chen Yan imagined emotional excess to obstruct the downward movement of *qi* in the body. This, in turn, resulted in stagnation of fluids, excess drool, and the formation of phlegm. As Harper (1998, p. 81) has shown, the earliest imaginations of *qi* flow in the body focused exclusively on this downward movement and considered its obstruction or reversal to be pathological.^12^ According to the *Inner Canon’s* logic of systematic correspondence, downward movement resonates with the autumn, which resonates with the Lungs, which govern the movement of *qi*. The Lungs, as we saw above, are the only organ system mentioned in the *Inner Canon* that is explicitly connected to disorders of *qi* constraint. All of the major herbs in Chen Yan’s two formulas are acrid, the flavor associated with the Lungs, and in the physiology of the *Inner Canon,* the Lung also plays a major role in moving water in the body (Porkert 1974).

This synthesis exerted a profound influence on later conceptions of emotion-related disorders. Most important, without doubt, is the etiological link that Chen Yan established between emotion-related disorders and pathologies of *qi* movement, including a clear understanding of how emotions produce physical symptoms that can be read off the body’s exterior. This was later summarized by the influential Qing dynasty physician Ye Tianshi’s 葉天士 (1667–1744):

Stagnation, whether present in the body or the organ systems must have visible manifestations of tension. *Qi* by its nature has no form but in the course of constraint the *qi* gathers together. This gathering together makes it appear to possess a form even if in reality it has no material substance. (Ye Tianshi 葉天士 1746, p. 173)

Chen Yan’s distinction between emotions as symptoms and emotions as causes, meanwhile, directly led to Zhang Jiebin’s 張介賓 (1563–1640) famous differentiation between “constraint caused by disease” (因病而鬱 *yīn bìng èr yù*) and “constraint causing disease” (因鬱而病 *yīn yù èr bing)* (Zhang Jiebin 張介賓 1624, p. 1124). Sharing a common point of origin and similar orientations Ye and Zhang, nevertheless, sometimes arrived at quite different conclusions. Zhang traced all emotion-related disorders back to the Heart organ system, while Ye emphasized the uniqueness of each clinical situation and favored a more flexible use of treatment strategies. Starting from Chen Yan’s own novel synthesis, they reached their different positions via the mediation of Zhu Danxi, whose ideas on constraint emerged in the context of a comprehensive critique of Chen Yan’s style of medical practice.

## Zhu Danxi 朱丹溪 (1281–1358)

Chen Yan most strongly influenced a group of physicians based in the Zhejiang city of Wenzhou, where he himself had also lived (Liu Shijue 劉時覺 2000). The title of one of their major works—*Simple Book of Formulas* 易簡方—hints at the ethos that tied together the various members of this group: namely, to devise simple and effective medical treatments with maximum benefit. Chen Yan had pursued this goal by establishing correct and, therefore, effective relationships between named diseases and their treatment. Explicit references to the “rectification of names” tied this project ideologically to Confucian models in the ancient past. A second and more immediate inspiration for Chen Yan and his followers was the Song government’s policy of promoting public welfare through the supply of effective drugs and formulas discussed above, particularly the official formularies.^13^ These formularies exerted considerable influence on medical practice during the Song and many of its formulas remain a mainstay of East Asian medicine to the present day. Yet, precisely because of this influence the formularies also attracted their fair share of critics. Beginning in the 12th century, the universalism underpinning this imperial vision of medicine was increasingly challenged by medical currents centered on distinctive masters and rooted in specific locales. These currents mirrored the emergence of diverse Neoconfucian schools of thoughts as well as a more general turn toward localism in the social life of the southern elite (Hinrichs 2003).

The most influential of these new style practitioners was Zhu Danxi, a Neoconfucian scholar turned physician who also lived in Zhejiang. One of Zhu’s most famous texts is a no holds barred attack on the Imperial Pharmacy’s collection of formulas and the style of medicine it stood for (Zhu Zhenheng 朱震亨 1347a). This critique emphasized two main points. First, it depicted the acrid moving drugs favored by the Imperial Pharmacy’s prescriptions as well as by physicians like Chen Yan and the Wenzhou current as potentially harmful to the specific needs and constitution of Zhu’s southern clientele. Second, it viewed the use of ready-made prescriptions as insufficiently sensitive to the diversity and contextual nature of all illness. Comprehensive in its breadth and depth, Zhu’s own approach focused on understanding disease dynamics rather than creating comprehensive nosological systems. He also emphasized an individual’s level of learning and self-cultivation rather than the words of the classics as the true foundation of clinical excellence (Furth 2006). His approach to the treatment of “constraint disease” (鬱疾 *yùjí*), another new term that appeared at the time, exemplifies this re-orientation even as it builds on Chen Yan’s earlier innovations.

Reiterating the centrality of flow expressed in *The Annals of Lü Buwei* and prefiguring the subsequent redefinition of stagnation as the cause of all disease in Edo Japan, Zhu Danxi defined constraint of *qi* and blood as capable of “generating [all of the] myriad disorders.” He explored obstructions to this flow through a new doctrine of “six [types of] constraint” (六鬱 *liǔ yù*) that over subsequent centuries came to rival and increasingly replace the five types of constraint outlined in the *Inner Canon*:

A hemming in of [the free flow] of the seven emotions, the combined invasion of cold and heat, continued exposure to rain and damp, or the build-up of alcohol [within the body], all of these produce constraint disorders. Furthermore, heat constraint producing phlegm, phlegm constraint producing cravings, food constraint producing focal distention and fullness, these are inevitable principles. (Zhu Zhenheng 朱震亨 1481, pp. 159–160)

Zhu’s focus on disease dynamics explains the widening of potential etiologies of constraint beyond the external causes emphasized in the *Inner Canon* and Chen Yan’s emotional causes to also include diet and lifestyle. Vis-à-vis Chen Yan, the pathophysiology of constraint is similarly expanded from the stagnation of body fluids associated with *qi* downward flow to now include also the generation of pathogenic heat, the accumulation of food, and stasis of blood. These innovations point to the increasing importance that post-Song physicians accorded to two hitherto relatively unimportant concepts: the “*qi* dynamic” (氣機 *qì jī*) and the role of fire (火 *huǒ*) in physiology and pathology (Ding Guangdi 丁光迪 1999).

The *qi* dynamic is a technical term in Chinese medicine that refers to the up/down, inward/outward movement of *qi* and body fluids. Owing to a wider interest in circular movements and rhythms that reflects influences from India and Buddhism (Despeux 2001) physicians’ attention to the *qi* dynamic extended existing preoccupations with *qi* downward flow toward a more comprehensive understanding of both ascending and directing downward (升降 *shéng jiàng*). This was coupled with a related shift toward fire as both an animating force of physiological process and a pathological agent. A key process by which fire as a pathogen was thought to arise in the body was through processes of stagnation and constraint.

*Escape Restraint Pill* 越鞠丸, a new herbal formula Zhu Danxi composed to guide the treatment of constraint, translates both of these theoretical re-orientations into concrete clinical practice. Its chief ingredients continue to be acrid and warming, which are the characteristics of medicinals that actively move the flow of *qi*, but they focus on the Spleen and Stomach systems rather than the Lungs as the new fulcrum of the *qi* dynamic; it contains bitter cooling herbs to drain heat from constraint; and it treats stagnation of food and blood as well as those related to *qi* and body fluids (Scheid et al. 2009, pp. 507–511). Furthermore, unlike the prescriptions contained in the Imperial Pharmacy’s formularies or those recommended by Chen Yan and his followers, *Escape Restraint Pill* was not intended as a specific remedy for treating a narrowly defined disease. Rather, it was conceived as a paradigmatic model guiding clinical practice with individual ingredients outlining possible strategies rather than denoting fixed constituents. It is this shift in thinking about the nature of named prescriptions that is perhaps the most emblematic signifier of the Neoconfucian transformation of medicine reflected in Zhu Danxi’s work.^14^

In Chinese, what we call “Neoconfucianism” is referred to as the “study of coherence” (理學 *lǐxué*) or the “study of the Way” 道學 (*daòxué*). These terms seek to convey a fundamental epistemic shift away from texts (文 wén) as repositories of exemplary models that serve to guide action in the present to one in which actions express an individuals’ own accomplishments and ability to grasp the true order of the world. Using Neoconfucian terms, the “human Heart” (人心 *rén xīn*) has the capacity to align itself with the “Heart of the Way” (道心 *daò xīn*), and it is out of this alignment that morally correct actions flow. Coherence (理 *lǐ*) refers to the fundamental but invisible patterning that produces the myriad manifestations of *qi* flow in the visible world (Bol 2008). In order to gain the insight necessary for grasping how coherence manifests through *qi* at any one moment in time, physicians at the bedside, like scholars seeking to make correct moral and political choices, had to cultivate their own Heart so that it would spontaneously mirror the Heart of the Way. Fixed prescriptions or the words of the sages could never accomplish this by themselves. In practice, they had to be reinvented again and again to cohere with the unfolding order of things that in the medical domain become intelligible through specific constellations (證 *zhèng*) of symptoms and signs at the bedside (Volkmar 2007). Accordingly, from then on, physicians were encouraged to modify and substitute formulas based on individual presentations to be considered true scholar physicians.

Zhu Danxi’s approach to constraint articulated existing therapeutic repertoires to newly emergent perceptions of *qi* flow and conceptions of effective practice. Compared to his immediate predecessors it downplayed the role of emotions by considering them to be just one of many possible causes. This did not indicate a lessening of interest into emotions per se, however. Rather, Zhu drew on another emergent theme of post-Song medicine, that of yin fire (陰火 *yīnhuǒ*), to tie together the role of emotions in the etiology of disease with their wider roles in human life. To this end, he extended the function of physiological fire in the body as a source of change and transformation to the all-important task of aligning the human Heart with the Heart of the Way. Yet, precisely because fire—visible in desire and emotional expression—animated life so comprehensively, if it burned out of control, it could easily turn into the most destructive of all pathogens. Controlling physiological fire in order for it not to become pathogenic yin fire thus became an important new task of post-Song medicine.

For Zhu Danxi this was first and foremost an issue of morality and the proper conduct of one’s life so as not to inflame desire and stir the emotions. As a physician familiar with human frailty, he was equally ready, however, to provide pharmacological solutions. This could involve draining fire arising from constraint. More often and more importantly, it meant draining excess fire from the (human) Heart and tonifying Kidney essence, a substance that controlled physiological fire but was readily consumed by yin fire. Zhu Danxi also associated the management of physiological fire in the body with the Liver organ system—indeed, his discussion provided the *locus classicus* for all later physicians—but he did not accord it a primary role in treatment either of constraint or yin fire.

As a teacher and writer, Zhu exerted enormous influence on the development of medicine throughout East Asia for centuries to come. These ideas continue to shape experience of bodily and personal unease across East Asia even today as in the case of *hwa*-*byung* in Korea described by Suh. Yet, reconciling the two interrelated yet different ideas regarding the relationship between *qi* flow and pathogenic fire within his larger oeuvre—an older one visible in his ideas about constraint and a newer one dominating his discourse on yin fire, desire, and the emotions—represented a considerable challenge to his successors.^15^ It led, on the one hand, to a redefinition of constraint as a purely emotion-related disorder and, on the other, to a re-evaluation of the Liver as the most important organ in the human body.

## Making Constraint Emotional

From the 15th and 18th centuries, a “cult of emotion” swept through elite Chinese culture (Santangelo 2003, 2005). This cult emerged at the confluence of many different factors. Leading intellectuals in Ming China had gradually moved the discourse on emotions away from negative associations with selfish desires in need of control to experiences that might be valorized as a source of knowledge and insight, and even as the foundation of truly human social bonds. Rapidly rising levels of literacy and a booming publishing industry created a market for the production and consumption of emotionally charged literary works. Many of these books were authored by educated women, for whom writing and reading provided a socially acceptable outlet for their creativity but eagerly consumed by readers of both sexes. Many of their male readers were frustrated literati whose advancement was blocked by corruption at all levels of the bureaucracy within a system endemically short of opportunity. The cult of emotions presented these men with new opportunities to reassert their elite status through a display of cultural sophistication. Members of the increasingly powerful and influential merchant class, meanwhile, sought to gain social acceptance by internalizing literati aesthetic ideals and values. Precisely these men and women, as we have seen, constituted the clientele of elite physicians in southern China.

Not surprisingly, a new nosological category of “emotion-related disorders” (情志病 *qíngzhì bing*) emerged in medical writings of the time of which constraint formed but one part (Messner 2000, 2006). A pivotal person driving this process was Xu Chunfu 徐春甫 (1520–1596) from Anhui province, then a main center of commerce and medical innovation. Xu’s main work, the *Systematic Great Compendium of Medicine Past and Present* 古今醫統大全 published in 1557, contains a chapter on constraint that defined its etiology for the very first time in purely emotional terms:

“Constraint is a disorder of the seven emotions. Therefore eight or nine out of ten patients suffer from it … Chronic constraint manifests in innumerable types of disease. Men who have it become deficient and cowardly or manifest with dysphagia occlusion, *qi* fullness or abdominal distension. Women who have it stop having their periods, or manifest with miscarriage, uterine bleeding, or deficiency taxation. Treatment strategies must be able to interiorly nourish, before opening constraint and regulate according to the presenting constellation [of symptoms and signs]” (Xu Chunfu 徐春甫 1557, p. 211).

Xu’s account contains several important re-articulations of existing ideas. First, while previously constraint tended to be seen as an acute blockage of *qi* movement, Xu argued that in his own time it manifested mainly as a chronic condition. This necessitated a change in treatment strategies from moving *qi* with acrid warming medicinals toward nourishing deficiency by means of sweet flavors. Second, while Zhu Danxi and his followers had clung to prevailing cultural sentiments that viewed women as especially prone to suffering from emotion-related disorders, Xu took the un-gendering of constraint begun by Chen Yan to its logical conclusion when he explicitly defined men as being equally at risk.

Xu Chunfu’s status as a court physician ensured rapid and wide exposure for of his ideas. *The Systematic Great Compendium* was reprinted in 1570, 13 years after its original publication, and its definition of constraint can be found almost verbatim in the work of later authors. Over the next century, many physicians attempted to accommodate Xu’s innovations with the earlier conceptions of constraint put forward in the *Inner Canon* and the writings of post-Song revisionists like Zhu Danxi. Participants in these debates disagreed with each other about virtually all of the points raised by Xu: whether constraint was purely emotional; to what extent it was caused by deficiency; whether it was primarily a woman’s disorder; and how it might best be treated.

Several authors explicitly cautioned against excessive reliance on drug-based treatments for emotional disorders, advocating long-established alternatives like emotional counter-therapy instead.^16^ Miao Xiyong 繆希雍 (1546–1627), from neighboring Jiangsu, went one step further. He opined that even if medicinals might successfully open up the movement of *qi* and blood, the condition would relapse if the “Heart disorder” 心病 at its root was not resolved. In this context, the term Heart disorder harks back to older views of the Heart as governing emotions, equating to what nowadays we might describe as cognitive and affective disorders. Any such disorder did not require herbs or minerals but “Heart medicinals” 心藥, defined by Miao as “the use of thought to change thought” 以識遣識 and “the use of reason to transform emotions” 以理遣情 (Miao Xiyong 繆希雍 1625, p. 32). Although no clear line of transmission can be made out, this approach closely resembles that of Wada Tokaku 和田東郭 (1744–1803) in Edo Japan discussed by Daidoji (this issue).

In China itself, the individualized and highly competitive nature of elite medicine worked against a resolution of these differences. As Karchmer (this issue) shows, it required the intervention of the state into the medical domain to bring this about. Yet, the synthesis achieved in the 1950s and 60s was crucially dependent on the innovations of late Ming medicine. Beyond tying emotions to constraint more closely then ever before, these also included the novel articulation between constraint and the Liver as the most important organ in internal medicine. Like everything else, this articulation was not posited as a hypothesis to be accepted or refuted at a definitive moment in time, but gradually emerged through a series of interlinking events.

## Making Fire Central

We previously saw that Zhu Danxi posited constraint and yin fire as two of the most crucial issues in internal medicine but did not tie them together into a single pathology. This was left to Zhao Xianke 赵献可 (n.d.), who lived in Ningbo, a wealthy port city in Zhejiang Province some two hundred miles up the coast from Wenzhou, at the turn of the 17th century.^17^ Zhao is counted today as belonging to a group of Ming dynasty physicians who further developed post-Song concerns regarding the importance of physiological fire, but rejected Zhu Danxi’s strategies for draining its pathological excess. Instead, these physicians advocated to support and nourish this fire and to facilitate its diffusion through the body with warming and supplementing medicinals. Quite naturally, this led Zhao to become concerned about constraint impairing the diffusion of physiological fire. Foreshadowing the programmatic return to the classics that would soon come to dominate medical discourse in China and Japan, he (re-)turned to the *Inner Canon* itself for inspiration:

In my opinion all disorders can arise from constraint. Constraint has the meaning of something being restrained and blocked. The [original] strategies in the *Inner Canon* were concerned with constraint arising from seasonal *qi* riding [the body’s own *qi*]. They were definitely not concerned with constraint due to worry, where worry implies disorders of the seven emotions, though worry also comes under [the wider category of constraint]. (Zhao Xianke 趙獻可 1687, p. 55)

It was this turning back to the classics that led Zhao into a very different direction than many of his contemporaries, namely toward emphasizing once again the role of external pathogens in the generation of constraint while dismissing the primary role of the emotions. Yet, like all commentators before and since, Zhao approached the *Inner Canon* very much with his own agenda in mind. For him, this was to understand and treat the (patho-)physiology of physiological fire, the force that for Zhao Xianke even more than for Zhu Danxi constituted the source of all life and creativity within the human body/person. Using the symbolic language of the five phases, Zhao likened this force to “the formless fire within wood,” the endless creativity of spring that manifests in upward-tending growth, but is easily constrained by adverse conditions. Therapeutically, this fire had to be nourished and any constrained to be removed. The key formula he selected for this purpose was *Rambling Powder*, first listed in the Song dynasty Imperial Pharmacy’s *Formulary* as a prescription for treating manifestations of feverishness associated with problems of menstruation. Zhao now significantly reinterpreted the actions of this prescription. Instead of a formula for women’s disorders, he praised it’s ability to promote physiological fire to spread moderately throughout the body, and thus as the “one method that can replace the five methods” for treating constraint originally listed in the *Inner Canon*. (Zhao Xianke 趙獻可 1687, p. 56).

*Rambling Powder*, like all other major formulas advocated by Zhao, is primarily a supplementing formula. Unlike Chen Yan and Zhu Danxi’s strategies for resolving constraint by means of harsh acrid and warming medicinals, it, therefore, unblocked stagnation in a gentler manner:

Within this formula the [combination of] the two herbs thorowax root and peppermint is the most marvelous. … To use an analogy, at the time of year when the first shoots emerge but have not yet grown a cold wind will constrain all [future growth] causing the plants to wilt as their [*qi* is] hemmed in, unable to extend upwards. … But it only takes a breath of warm wind for the constrained *qi* to smoothly flow again … Thorowax root and peppermint are acrid and warming. Being acrid they are able to emit and disperse. Being warming they enter the lesser yang [channel]. This shows the marvel of the ancients in composing formulas. (Zhao Xianke 趙獻可 1687, p. 57)

Needless to say, such gentle treatment resonated not only with more stereotypical images of women, for whom *Rambling Powder* had originally been composed, but also with the new gender identities that had developed among southern China’s “fragile scholars.” Their deficiencies were further supported by Zhao’s advice to follow up the use of *Rambling Powder* with *Six*-*Ingredient Pill with Rehmannia* 六味地黃丸, a well-known Song dynasty formula for strengthening Kidney essence: the physiological source of fire within wood. Here, in spite of any professed difference, Zhao Xianke converged on Zhu Danxi, who, as we saw, also advocated supplementing the Kidneys in order to nourish and control fire. Zhao’s use of *Rambling Powder* as a prescription to be used with minimal modifications for all cases on constraint, on the other hand, represented a considerable simplification of medical practice reminiscent of the prescription-based approach advocated by Chen Yan and the Imperial Pharmacy’s Formulary, from which *Rambling Powder* was, of course, taken.

Undoubtedly, it was the simplicity of this therapeutic approach that made it so attractive to physicians for generations to come. Over time, as we saw in the “Introduction” section, they simplified that approach even further. First, they stripped it of its physiological basis (namely, perceiving of constraint as an obstruction of physiological fire) to narrow its range of indications once more to emotion related constraint disorders while forgetting Zhao’s own concern with externally contracted pathogens. Second, by interpreting “fire within wood” as being all about the Liver organ system rather than the diffusion of physiological fire they could focus on one organ rather than a complex set of physiological functions. At the time, however, the wide-spread adaptation of these simplifications was far from predetermined.

## Constraint as Pathology Versus Constraint as Disease

A key role in this process was played by Ye Tianshi 葉天士 from Suzhou in Jiangsu Province, the leading center of medical innovation between the 17th and 19th centuries. Ye was a brilliant synthesizer, able to fuse the competing approaches of pre- and post-Song medicine into a flexible style of practice that focused on treating presenting patterns rather than named diseases in the manner advocated by Zhu Danxi. This style of practice could not be learned by memorizing formulas. It had to be assimilated slowly in the course of personal apprenticeships or deduced with much effort from studying the case records of famous physicians. Many of Ye’s own cases were, therefore, compiled by students and admirers into the enormously influential *Guide to Clinical Practice Based on Patterns* 臨證指南醫案, which made him the most famous physician of his time. Yet, the synthetic approach that underpinned Ye’s own versatility became a crucial node in the simplifications that today tie constraint to the Liver, *Rambling Powder*, and finally depression.

Ye’s *Guide to Clinical Practice* contains a chapter on constraint that acknowledges the wide range of meanings the term had acquired over previous centuries. He resolved the tension between competing definitions by distinguishing between constraint as a patho-physiological process and constraint as a named disorder, a differentiation first made by Zhang Jiebin in the late Ming, and harking back to Chen Yan in the Song. Constraint as a patho-physiological process could occur in the course of all kinds of disorders, including those involving the contraction of external pathogens. Constraint, as a named disorder, was primarily an emotion-related condition that required for the patient to develop insight into the root causes of the problem and to change his or her behavior accordingly. For Ye himself, pharmaco-therapy could only ever be an adjunctive treatment. Such treatment had to be flexible, uniquely adjusted to each individual patient, sometimes nourishing, sometimes moving, but always gentle and never excessively reliant on harsh medicinals for fear of exciting rather than moderating the diffusion of physiological fire. Constraint as a patho-physiological process is much more common in Ye’s *Guide to Clinical Practice*. Occurring altogether 736 times in the text, it refers to stagnation of *qi* and fluids due to multiple causes and the consequences thereof and requires an even wider range of treatment strategies.

Ye pushed these strategies even further in their focus on gentleness, not only in the treatment of constraint, but across the entire spectrum of internal and external medicine. Wherever possible, he even avoided thorowax root—favored by Zhao Xianke as a gentle alternative to medicinals such as magnolia bark or nutgrass rhizome used by Chen Yan and Zhu Danxi—because he considered its effect on opening up the *qi* flow as potentially leading to the exhaustion of Kidney essence, the deepest root of person’s vitality. Not surprisingly, when from the late 19th century onward China once more looked to sturdier men and women to undertake the work of modernization and catching up with the West, Ye Tianshi’s followers were widely decried as ineffective “peppermint doctors.”

## The Liver as the Most Important Organ

Ye Tianshi’s influence on the development of Chinese medicine extends to many other areas, including new ideas for thinking about Liver disorders. Ye employed the idea that the Liver was the organ in charge of managing physiological fire first posited by Zhu Danxi in order to tie together different types of *qi* pathologies that physicians hitherto had found difficult to reconcile with each other. To this end, he argued that problems of *qi* stagnation, (yin) fire and wind (another important disease category in Chinese medicine) were nothing but different manifestation of Liver disorders. Without going into the finer details of this synthesis, Ye thereby succeeded in elaborating a single framework for thinking about *qi* constraint and physiological fire, the two strands of Zhu Danxi’s thinking that had first established a truly “southern” medicine. Unlike Zhao Xianke, who had attempted a similar synthesis, Ye did not think of these problems as essentially constituting one type of disorder, and he continued to advocate individually specific prescription. To resolve stagnation and constraint, for instance, Ye placed equal importance on facilitating upward diffusion of fire governed by the Liver and on the downward moving of *qi* and body fluids governed by the Lungs. Yet, once in place, his doctrine lent itself to various kinds of simplifications that turned the Liver into the single most important focus of internal medicine.

From the mid 18th century onward, this new new focus on the Liver swept through medical circles in southeast China (Lu Yitian 陸以湉 1858). The Suzhou physician Wang Tailin 王泰林 (1923), for instance, detailed thirty different strategies for treating the Liver, more than were known for any other organ system. Huang Yuanyu 黃元御 (1705–1758), who stemmed from Shandong but practiced in the southeast, claimed that the Liver system was involved in 80–90 % of illnesses he saw in practice (Huang Yuanyu 黃元御 pref. 1753, p. 40). Most importantly, the Zhejiang physicians Wei Zhixiu 魏之琇 (1722–1772) and his popularizer Wang Shixiong 王士雄 (1808–1868) defined the Liver as the root of all emotion-related disorders, a connection that as we learned in the “Introduction” is repeated today as a statement of fact within TCM circles.

“The Lungs govern the exterior of the entire body, while the Liver governs its interior. The contraction of the five [climatic] *qi* [from the exterior] thus proceeds via the Lungs, while disorders of the seven emotions arise of necessity from the Liver. This is something about which I have spoken at length. Master Wei [Zhixiu] excels in internal damage and his words touched my heart early on.” (Wang Mengying 王孟英 1851, p. 882)

By the mid 20th century, when physicians in China found it necessary to define themselves with reference to the West, the Liver even assumed nationalist dimensions in the publication of Zhao Shuping’s 趙樹屏 (1931) *Treatise on Liver Disorders* 肝病論. The title of this treatise self-consciously referred back to *Treatise on Cold Damage*, the foundational text of Chinese pharmacotherapy newly in vogue at the time because of its apparently empiricist orientation, arguing that Liver disorders were most important in China while Lung disorders where more typical of Westerners.

Even more interesting than the movement of the Liver to the center stage of medical practice in late imperial China is how little controversy and debate attached to it. Lu Yitian (1858) suggests a lineage of ideas extending from Zhao Xianke to Wei Zhixiu via Ye Tianshi, but otherwise physicians and medical historians alike have been largely silent on this movement. Such silence is extremely unusual in any living tradition. It certainly is not the norm for Chinese medicine. Southern physicians were criticized by their peers and by physicians in Republican China for putting the Lungs at the center of externally contracted warm pathogen disorders, for not sticking to orthodox interpretations of *the Treatise on Cold Damage*, for their choice of mild acting medicinals, and for much else besides. No one, however, seems to have raised similar concerns about just why the Liver was suddenly accorded an importance that it lacked for the previous fifteen hundred years, and why, in spite of the many different views outlined here, it should so suddenly become the key to treating all emotion-related disorders.

One reason, as Ernest Renan reminds us in the epigraph at the start of this paper, is that any tradition must forget part of its own history in order to allow it to share certain ideas. To construct a national medicine, as Chinese physicians have attempted for the last century, the southern origins of what had by then become the dominant style of medical practice had to be downplayed even as they became a mainstay of modern TCM.^18^ In the concluding section, I will use this insight to explore what the genesis of constraint as an emotion-related Liver disorder outlined in this essay may be able to tell us about history of medicine in East Asia and the articulation between body and emotions at large.

## Conclusions

In the same sense that ICD and DSM definitions of depression and a host of other psychiatric disorders are said to reflect specific Western experiences of body, person and self, the articulations between constraint as a emotion-related disorder, the Liver and the gentle treatment strategies of formulas such as *Rambling Powder* embody the sensibilities not of “the Chinese” but of a distinctive southern Chinese elite in late imperial China. These sensibilities were formed at the interface of changing epistemic preoccupations, gender identities, and the flesh and blood of human existence. This article has charted, at least in outline, the genesis of this articulation. It was this historically specific articulation, and not ancient Chinese wisdom, that was connected—again under very specific conditions of emergence—to Western ideas about emotion-related disorders as nervous system disorders in the early 20th century (Karchmer, this issue). In Korea and Japan and also in other parts of China, such linkages were constructed via different routes even if they, too, had some of their roots in the medicine of Chen Yan, Zhu Danxi, or Zhao Xianke (Daidoji and Soyoung, this issue).

One telling example of such difference is the use of the formula *Separate the Heart Qi Drink* 分心氣飲. Like *Rambling Powder* first listed in the Song Dynasty Imperial Pharmacy’s *Formulary*, its original indications included, “any disharmony of *qi* in both men and women … due to worry, [excessive] thinking, or angry *qi* damaging the spirit, worrying while eating, or affairs not proceeding as intended causing the constrained *qi* to stagnate without dispersing…” (Editorial Committee of the Great Encylopedia of Chinese Medicine 中醫大詞典編輯委員會 1983). *Separate the Heart Qi Drink* is still commonly prescribed to treat “*qi* stagnation due to the seven emotions” in Korea and Japan, but few physicians in contemporary China even know of its existence.^19^ In their university textbooks, those Chinese physicians, furthermore, learn that *Escape Restraint Pill*, quite contrary to Zhu Danxi’s own ideas, is a formula for resolving Liver *qi* constraint, while *Rambling Powder*’s associations with external disorders are all but forgotten.

Which conveniently leads us back to where we began this journey: namely, to the multiple tensions between Zhang’s definition of constraint as intelligible only within the discourse of Chinese culture, Ng’s unsuccessful attempts at universalizing it as a biomedical psychiatric disease category, and the de facto globalization of constraint within TCM practice. This globalization has allowed constraint to escape its more narrow attachments to Chinese culture and language. It succeeded where Ng failed because it found a way to circumvent the dominant techno-scientific-bureaucratic networks that he accepted as key to such global diffusion. Yet, such diffusion could not happen without TCM practitioners positioning themselves as offering hope to those whom psychiatry has failed or who look for alternatives to biomedicine in their personal quest for health. As we saw in the introduction, this implies creating equivalences between terms such as “depression” and “Liver *qi* stagnation,” and to make stress intelligible as constraint. Even as it promises an alternative, this strategy of translation ends up supporting the reality of biomedical psychiatry by merely inserting TCM practice within it. As a result, the Cochrane collaboration, which we can assume has not the slightest understanding of the Liver organ system in Chinese medicine nor believes in the reality of *qi*, has recently expressed interest in evaluating the effectiveness of *Rambling Powder* in the treatment of depression.

Different as they may seem, contemporary TCM practices, Ng’s attempt to capture the essence of constraint through a list of symptoms and even Zhang’s definition of constraint as embodying enduring Chinese aesthetics of self-fashioning thus work toward a similar endpoint: the condensation of two thousand years of contested history into a something more simple and manageable. One might lament a certain loss of authenticity or critique the simplifications involved. One can also view these efforts as merely another stage in the ongoing transformations of constraint. If previously these had been shaped by changing epistemic and lifestyle formations, first in southeast China and then in East Asia, the globalization of constraint simply forces it to articulate with new technologies, institutions, concepts, and practices including those originating in the West. Simplification, furthermore, appeared as a recurrent feature in the process of genesis outlined here: Chen Yan’s erasure of male/female differences in formula indications; Xu Chunfu’s designation of constraint as invariably emotional in origin; Zhao Xianke’s discovery of the one strategy that could replace the five strategies of the *Inner Canon*. In fact, one could argue that it is precisely by way of such simplification that ideas and practices persist and travel across space and time. Creating equivalences between depression and constraint was simpler, in the end, than adding constraint as a new disease to DSM and this is why TCM succeeded where Ng did not.

However, all simplifications must eventually fail, and that, too, is a recurrent theme in the story outlined here. Old ideas and practices no longer fit new ways of being in the world. Treatment strategies for constraint in post-Song China turned increasingly away from harsh acrid medicinals toward gentle and mild acting ones that resonated with the manner in which southerners, and more specifically the southern gentry elite, embodied constraint. It is apt, therefore, to end my discussion with reflections on the use of *Rambling Powder* from the blog of “Acupuncture Carl,” an American acupuncturist and herbalist living in Tokyo. Commenting on changes in the body/person of men who take up Vipassana meditation, he writes:

Interestingly, as men adopt the Vipassana lifestyle […] they seem to become prime candidates for *Rambling Powder* when their yin and yang go out of whack. Does this mean that Vipassana makes men more feminine? This might be assumed since they are now more likely to be *Rambling Powder* candidates, but it seems that by reducing yang, the meditator’s lifestyle leads to a less aggressive male, though not a more feminine one (Stimson 2010).

To the average person this quite likely reads like so much new-age thinking. At the end of this discussion, however, Stimson appears to be a rather astute observer of something more profound: the constitution of body/persons at the always emergent interface of biology and culture. Lock and Kaufert (2001) refer to these living articulations as “local biologies.” A similar notion of the interrelated “shape or disposition of human process” (Ames 1984) is implicit, too, in various Chinese terms for body like shēn 身 (the lived body) and tǐ 體 (embodiment). Body/persons (shēntǐ 身體) are malleable but the malleability of local instantiations is constraint by shared universals. Hence, doing similar things, Stimson’s meditating men and Neoconfucian literati end up embodying similar body/persons. The difference is that whereas Confucian scholar physicians had to construct effective interventions for these body/persons by articulating various strands of their tradition in innovative ways, Stimson could fall back on an already existing repertoire of potentially appropriate responses. The modern articulation of constraint to biomedical disease categories like depression is a similarly creative response to the ever-changing embodiments of illness. However, as the historical account of this genesis demonstrates, it is man-made and not a simple matter of fact. It contains insight but also reflects biases and the effects of power. For while most Chinese medicine physicians accept the reality of depression, few psychiatrists extend the same respect to constraint. Unlike Acupuncture Carl, whose idiosyncratic musings are born entirely from his own observations, the TCM articulations between depression and constraint thus reflect a more complex history and set of power relations. To uncover these relationships it is necessary to unpack the simplifications that construct facts. Such unpacking allows us to view alternative possibilities and trajectories along which things may have unfolded. Read in this way, the history of constraint might facilitate more than mere insights into East Asian medicines and their integration into contemporary health care. It has the potential of deciphering relationships between body, mind and emotions that are not shackled by any single conception of the body/person or any single way of engaging with them therapeutically. Whether or not we wish to perceive these relationships and how we might chose to act upon them when we do is, of course, another question.

## Footnotes

1 “Emotion-related disorders” is Zhang’s translation of the Chinese term *qīngzhì bìng* 情志病. I use it throughout this discussion to refer to disorders that involve emotions in a significant manner as perceived by the actors I describe. Such involvement may pertain to causes, manifestations, or both. I use the term to avoid culture-laden terms such as “psychosomatic,” whereas the more simple “emotional” tends to highlight expression over causation.

2 Chinese medicine explains the functioning of the human body with reference to various organ systems. Although these organ systems share their names with the anatomical organs of biomedicine, they are not the same. For this reason I follow the convention to capitalize the name of these organ systems to thereby differentiate them from the anatomical organs of biomedicine. As this paper demonstrates, ideas about the precise nature and function of these organ systems change over time. However, there is a shared perception that these organ systems have functions that extend across biomedical, anatomical, and physiological boundaries. For instance, the Liver organ system reflects in the condition of the nails and muscle-sinews and expresses itself in emotions such as anger or sadness.

3 These translations are based on the understanding that the characters 逍遙 refer to the title of the first chapter of the Daoist classic Zhuang Zi 莊子, “Rambling Without a Destination,” which includes many stories about soaring above a restricted world view.

4 Interestingly, however, prescribing practices in Australia seem to be quite different based on data supplied to me by Helios Supply Company (Chianese 2011)

5 A search of Chinese medical journals between 1986 and 2011 using the search terms “subject = ‘逍遙散’ AND ‘抑鬱症’” carried out on 27 May 2011 using the China Academic Journals database produced 145 hits. Similar searches carried out for other popular formulas produced the following number of hits: *Liquorice, Wheat and Jujube Decocotion*甘麥大棗湯 70 hits; *Return Spleen Decoction* 歸脾湯, 30 hits; *Pinellia and Magnolia Bark Decoction* 半夏厚樸湯, 26 hits; *Frigid Extremities Powder* 四逆散, 20 hits.

6 Note that Hedfors argues that Fleck’s own understanding of this historical genesis is extremely superficial and badly researched.

7 In fact, until the Song, climatic factors like cold and wind were regularly grouped together with emotions under the rubric of the “seven qi” 七氣. Chen Yan’s redefinition of these seven qi as emotions is thus another aspect of his redefinition of Chinese medicine based on etiological factors.

8 See also Hanson (2011) for a more complete account. Interestingly, although Hanson explores constitutional explanations in Chinese medicine based on region and social class, she does not explore gender issues.

9 Unschuld (2003, p. 227) specifically cites Kleinman (1980, 1986) in support of this opposition.

10 Seven *Qi* Decoction 七氣湯 appears for the first time in the *Formulary of the Pharmacy Service for Benefiting the People in an Era of Great Peace* 《太平惠民和劑局方》. It can be seen as a variation of the formula *Pinellia Powder or Decoction* 半夏散幾湯 from the *Treatise on Cold Damage*, to which *ginseng* 人參 has been added (Li Peisheng and Liu Duzhou 李培生 1987, p. 489). Major Seven *Qi* Decoction 大七氣湯 is listed in the *Essentials from the Golden Cabinet* 金櫃要略 section of the *Treatise on Cold Damage and Miscellaneous Disorders* as *Pinellia and Magnolia Decoction* 半夏厚樸湯 (Scheid et al. 2009, pp. 516–519).

11 The main herbs in these formulas are *Pinelliae Rhizoma preparatum (zhì bàn xià)* 制半夏 and *Magnoliae officinalis Cortex (hòu pò)* 厚樸, both of which are acrid, warming and moving to break up stagnation of qi, fluids, and phlegm (Bensky et al. 2004, pp. 470–473).

12 This may be related to the close resonances between the concept of physiological *qi* and water and pathological *qi* and wind. For in nature, water always flows downward and so do food and other substances in the body, while wind is stronger at greater heights.

13 These were the *Revised Formulary of the Imperial Pharmacy* 《校正和劑局》 published in 1076, and the *Formulary of the Pharmacy Service for Benefiting the People in an Era of Great Peace* 《太平惠民和劑局方》, published in 1107 and reprinted in 1151.

14 In fact, the source text lists various herbal combinations for different types of constraint and only at the very end of the discussion lists *Escape Restraint Pill* itself. Zhu’s critique of fixed formulas is formulated in Inquiry into the Propensities of Things 《格致余論》(Zhu Zhenheng 朱震亨 1347b, pp. 43–44).

15 The former is associated primarily with the work of Liu Wansu 劉完素 (ca. 1110–1200), the latter with that of Li Dongyuan 李東垣 (1180–1251), both of whom influenced Zhu Danxi (Ding Guangdi 丁光迪 1999, p. 469). For a discussion of the importance of fire in post-Song medicine see also Plant (2011).

16 The influential *Investigations on Medical Formulas* 《一方考》, for instance, a monograph on the principles underlying formula composition written in 1584 by Wu Kun 吳崑 (1551–1620), who hailed from the same area of Anhui province as Xu Chunfu, contained a section on emotions 情志 that states: “When emotion is overwhelmingly excessive, no medicinals can cure [the resulting disorder]. It must be overcome by emotion. Thus it is said that anger damages the Liver system but sorrow overcomes anger; worry damages the Spleen system but anger overcomes worry; sorrow damages the Lung system but joy overcomes sorrow; apprehension damages the Kidney system but worry overcomes apprehension. A single saying from the *Inner Canon*, and a hundred generations have venerated it. These are immaterial medicines” (Wu Kun 吳昆 1584, p. 85). Wu Kun listed eleven anecdotes from the history of Chinese medicine describing the application of emotional counter-therapy when more conventional methods had failed to make it’s case.

17 I wish to acknowledge my gratitude to Leslie de Vries for considerably clarifying my understanding of Zhao Xianke’s approach to medicine.

18 As I show in my ethnography of Chinese medicine in contemporary China the establishment of TCM in the 1950 and 60s was dominated by physicians from Jiangnan, specifically Nanjing (Scheid 2007).

19 A search of Chinese medical journals between 1986 and 2011 using the search terms “abstract” = “分心氣飲” carried out on 12 December 2011 using the China Academic Journals database produced 20 hits, of which half were for discussions of the formula’s usage in Korea, Japan, and Vietnam. A similar search for *Rambling Powder* produced over 12,000 hits.

## References

[CR100] Ames, Roger T. 1984 The Meaning of the Body in Classical Chinese Thought. International Philosophy Quarterly 24(1):39–54.

[CR1] Baldrian-Hussein Farzeen (1996). Taoist Beliefs in Literary Circles of the Sung Dynasty—Su Shi (1037–1101) and His Techniques of Survival. Cahiers d’Extrême-Asie.

[CR2] Bensky Dan, Clavey Steven, Stöger Erich (2004). Chinese Herbal Medicine: Materia Medica.

[CR3] Bol, Peter Kees 2008 Neo-Confucianism in History. *In* Harvard East Asian Monographs 307. Cambridge, MA: Harvard University Asia Center (distributed by Harvard University Press).

[CR4] Butler, Lee and Karen Pilkington 2011 Chinese Herbal Medicine and Depression: A Systematic Review. School of Life Sciences. Unpublished presentation, University of Westminster, Westminster.

[CR5] Castle, Michael 2011 Personal Communication. V. Scheid (29 October 2011). Boulder.

[CR6] Chen, Patrick 2011 Personal Communication. V. Scheid (26 May 2011). London.

[CR7] Chen Yan 陳言 1173 Discussion of Illnesses, Patterns, and Formulas Related to the Unification of the Three Etiologies《三因極一病證方論》. *In* Wang Xali 王象禮, ed. Beijing: Zhongguo zhongyiyao chubanshe, 2005.

[CR8] Chianese, Tony 2011 Personal Communication. V. Scheid (20 December 2011). London.

[CR9] Deng Tietao 鄧鐵濤, ed. 1987 Chinese Medical Diagnosis 《中醫診斷學》. Beijing: Renmin weisheng chubanshe.

[CR10] Despeux Catherine, Hsu E. (2001). The System of the Five Circulatory Phases and the Six Seasonal Influences: A Source of Innovation in Medicine under the Song (960–1279). Innovation in Chinese Medicine.

[CR11] Ding Guangdi 丁光迪 1999 A Critical Analysis of Jin-Yuan Medicine 《金元醫學評析》. Beijing: Renmin weisheng chubanshe.

[CR12] Editorial Committee of the Great Encylopedia of Chinese Medicine 中醫大詞典編輯委員會, ed. 1983 Great Encyclopaedia of Chinese Medicine: Formulas 《中醫大詞典:方劑》. Beijing: Renmin weisheng chubanshe.

[CR13] Elvin Mark, Feher M. (1989). Tales of Shen and Xin: Body-Person and Heart-Mind in China During the Last 150 Years. Fragments for a History of the Human Body. Part Two.

[CR95] Fleck, Ludwig (1980) Enstehung Und Entwicklung Einer Wissenschaftlichen Tatsache. Frankfurt/Main: Suhrkamp.

[CR14] Furth Charlott. (1999). A Flourishing Yin: Gender in China’s Medical History, 960–1665.

[CR15] Furth Charlotte (2006). The Physician as Philosopher of the Way: Zhu Zhenheng (1282–1358). Harvard Journal of Asiatic Studies.

[CR16] Goldschmidt, Asaf Moshe 2009 The Evolution of Chinese Medicine: Song Dynasty, 960–1200. *In* Needham Research Institute Series. London, New York: Routledge.

[CR17] Hanson, Marta E. 1998 Robust Northerners and Delicate Southerners: The Nineteenth-Century Invention of a Southern Medical Tradition. Positions 6(3):515–550.

[CR18] Hanson Marta E. (2011). Speaking of Epidemics in Chinese Medicine: Disease and the Geographic Imagination in Late Imperial China.

[CR19] Harper Donald John (1998). Early Chinese Medical Literature: The Mawangdui Medical Manuscripts.

[CR20] Hedfors, Eva 2006 The Reading of Ludwik Fleck: Questions of Sources and Impetus. Social Epistemology 20(2):131–161.

[CR21] Hinrichs, T. J. 2003 The Medical Transforming of Governance and Southern Customs in Song Dynasty China (960–1279 C.E.). PhD, Cambridge: Harvard University.

[CR22] Huang Yuanyu 黃元御 pref. 1753 Essential Origins of the Four Sages 《四聖心源》. *In* Huang Yuanyu’s 11 Medical Works《黃元御醫書十一種》 (Vol. 3). Ma Ruiting 麻瑞亭, Sun Qiaxi 孫洽熙, Xu Shufeng 徐淑鳳 and Xiao Fangqin 蕭芳琴, eds. Beijing: Renming weisheng chubanshe, 1990.

[CR23] Joswick, Diane 2010 The Liver and Liver Qi Stagnation. El Segundo: Internet Brands, Inc./Acufinder.com. https://www.acufinder.com/Acupuncture+Information/Detail/The+Liver+and+Liver+Qi+Stagnation, accessed December 15, 2010.

[CR24] Kleinman Arthur (1980). Patients and Healers in the Context of Culture.

[CR25] Kleinman Arthur (1986). Social Origins of Distress and Disease.

[CR26] Kuriyama, Shigehisa 1997 The Historical Origins of Katakori. Japan Review 9:127–149.

[CR27] Li Peisheng 李培生, and Liu Duzhou 劉渡舟, eds. 1987 Treatise on Cold Damage 《傷寒論》. Beijing: Renmin weisheng chubanshe.

[CR29] Liang Jinghui 梁景輝 2010 Pressure and Constraint Most Easily Damages the Liver. Treatment by Chinese Medicine Relies on “Free and Easy” 《壓力、鬱卒易傷肝 中醫治療靠「逍遙」》. http://tw.myblog.yahoo.com/jw!_b8Re.uGGAWrSLTzujXjbMgVwFU-/article?mid=648&prev=649&next=647, accessed December 15, 2011.

[CR30] Liu Shijue 劉時覺, ed. 2000 Research on the Yongjia Medical Current 《永嘉醫派研究》. Beijing: Zhongyi guji chubanshe.

[CR31] Lock M., Kaufert P. (2001). Menopause, Local Biologies, and Cultures of Aging. American Journal of Human Biology.

[CR33] Lu Yitian 陸以湉 1858 Medical Essasy from out-of-the-Way Cottage 《冷廬醫話》. Zhu Feichang 朱偉常, ed., Shanghai: Shanghai zhongyixueyuan chubanshe.

[CR34] Luo Zhufen 羅竹風, ed. 1997 Encyclopedia of Chinese Language: Reduced Size Edition《漢語大詞典: 縮印本》. Shanghai: Hanyu dacidian chubanshe.

[CR32] Lü Buwei, Knoblock John, Riegel Jeffrey (2000). The Annals of Lü Buwei: A Complete Translation and Study.

[CR35] Mcgechie, Duncan 2009 How Are Practitioners and Students of Chinese Herbal Medicine in the Uk Informed in Their Clinical Choices? An Analysis of the Use of Xiao Yao San 逍遙散 and Its Modifications. MSc Dissertation, School of Life Sciences, University of Westminster, London.

[CR36] Messner, Angelika 2000 Emotions in Late Imperial Chinese Medical Discourse: A Preliminary Report. Ming Qing yanjiu, pp. 197–215.

[CR37] Messner Angelika, Santangelo P., Guida D. (2006). Making Sense of Signs: Emotions in Chinese Medical Texts. Love, Hatred, and Other Passions: Questions and Themes on Emotions in Chinese Civilization.

[CR38] Miao Xiyong 繆希雍 1625 Commentary on the Divine Husbandman’s Classic of Materia Medica《神農本草經疏》. *In* Collected Medical Works of Miao Zhongchun《繆仲淳醫書全集》. Li Shunbao 李順保, and Chu Xuanren 褚玄仁, eds. Beijing: Xueyuan chubanshe, 1998.

[CR39] Mote Frederick W. (1999). Imperial China 900–1800.

[CR40] Ng S.M. (2006). Stagnation as a Distinct Clinical Syndrome: Comparing ‘Yu’ (Stagnation) in Traditional Chinese Medicine with Depression. British Journal of Social Work.

[CR41] Ng Siu-man (2011). Confirmatory Factor Analysis of the Stagnation Scale: A Traditional Chinese Medicine Construct Operationalized for Mental Health Practice. International Journal of Behavioral Medicine.

[CR42] Plant, Simon 2011 Xiao Yao San. Personal Communication. V. Scheid (16 May 2011). London.

[CR43] Porkert Manfred (1974). The Theoretical Foundations of Chinese Medicine: Systems of Correspondence.

[CR44] Qiao Mingqi 喬明琦 and Zhang Minyun 張惠雲 2009 The Study of Emotions in Chinese Medicine 《中醫情志學》. Beijing: Renmin weisheng chubanshe.

[CR45] Santangelo Paolo (2003). Sentimental Education in Chinese History: An Interdisciplinary Textual Research on Ming and Qing Sources.

[CR46] Santangelo Paolo (2005). Evaluations of Emotions in European and Chinese Traditions: Differences and Analogies. Monumenta Serica.

[CR47] Scheid Volker (2007). Currents of Tradition in Chinese Medicine, 1626–2006.

[CR48] Scheid Volker (2009). Chinese Herbal Medicine: Formulas and Strategies (2nd Enlarged Edition).

[CR49] Song Geng (2004). The Fragile Scholar: Power and Masculinity in Chinese Culture.

[CR50] Stimson, Carl 2010 Meditation Men and Xiao Yao San. *In* Acupuncture Carl. Tokyo: Blogger. http://acupuncturecarl.blogspot.com/2010/03/meditation-men-and-xiao-yao-san.html, accessed on December 15, 2012.

[CR51] Tan Kaiqing 譚開清, ed. 1998 Differentiation and Treatment of Emotional Disorders 《七情病辨治》. Beijing: Zhongguuo yiyao keji chubanshe.

[CR52] Unschuld, Paul U. 2003 Huang Di Nei Jing Su Wen Nature, Knowledge, Imagery in an Ancient Chinese Medical Text. Berkeley: University of California Press.

[CR53] Unschuld, Paul U., Hermann Tessenow, and Jinsheng Zheng 2011 Huang Di Nei Jing Su Wen: An Annotated Translation of Huang Di’s Inner Classic—Basic Questions. Berkeley: University of California Press.

[CR54] Volkmar, Barbara 2007 Die Fallgeschichten Des Arztes Wan Quan (1500–1585): Medizinisches Denken Und Handeln in Der Ming-Zeit. München: Elsevier.

[CR55] Wang Mengying 王孟英 1851 Liouzhou’s Medical Essays and Good Formulas《柳洲醫話》. *In* Wang Mengying’s Collected Medical Works 《王孟英醫學全書》. Sheng Zengxiu 盛增秀, ed. Beijing: Zhongguo zhongyiyao chubanshe.

[CR56] Wang Tailin 王泰林 1923 Determination and Treatment of Liver Disorders 《肝病證治》. *In* A Collection of Late Night Essays from West Stream Studio 《西溪書屋夜話錄》. Shanghai: Shanghai qianqingtang shuju 上海千傾堂.

[CR57] Wu Kun 吳昆 1584 Investigations of Medical Formulas 《醫方考》. Guo Junshuang 郭君雙, ed. Beijing: Zhongguo zhongyiyao chubanshe, 1999.

[CR58] Xu Chunfu 徐春甫 1557 Systematic Great Compendium of Medicine Past and Present 《古今醫統大全》. Beijing: Kexue chubanshe, 1998.

[CR90] Yanhua Zhang 2007 Transforming Emotions with Chinese Medicine : An Ethnographic Account from Contemporary China. Albany: State University of New York Press.

[CR59] Ye Tianshi 葉天士 1746 A Guide to Clinical Practice According to Patterns 《臨證指南醫案》. Huang Yingzhi 黃英志, ed. Beijing: Zhongguo zhongyiyao chubanshe, 1999.

[CR60] Yoshimasu Tōdō 吉益東洞 1747 Organon of Medicine 《醫斷》. *In* Medical Works and Case Histories from the Yoshimasu Family 《吉益氏醫論醫案》. Beijing: Xueyuan chubanshe, 2009.

[CR61] Zhang Baiyu 張伯輿, ed. 1988 Chinese Internal Medicine 《中醫內科學》. Beijing: Renmin weisheng chubanshe.

[CR62] Zhang Jiebin 張介賓 1624 Collected Treatises of Jingyue 《景岳全書》. Beijing: Renmin weisheng chubanshe, 1991.

[CR63] Zhang Ruhong 章如虹, Jin Dishen 金棣生, and Mao Shusong 毛樹松, eds. 1997 Chinese Medicine Protocols for Treating Diseases According to Patterns 《中醫病症治療常規》. Beijing: Kexue jishu wenxian chubanshe.

[CR64] Zhao Guoyang 趙國祥, ed. 2009 Zhao Qingli’s Regulating Treatment of Constraint Patterns with Case Records and Medical Essays 《趙清理鬱証調治與醫案醫話》. Beijing: Renmin yunyi chubanshe.

[CR65] Zhao Shuping 趙樹屏 1931 On Liver Illness 《肝病論》. Lu Zheng 陸拯, ed. Hangzhou 杭州: Zhejiang Science and Technology Press 浙江科學技術出版社, 2003.

[CR66] Zhao Xianke 趙獻可 1687 Thread through Medicine 《醫貫》. Beijing: Xueyuan chubanshe, 1996.

[CR67] Zhu Zhenheng 朱震亨 1347a Elaborations on the Pharmacy’s Formular 《局方發揮》. Beijing: Renminweisheng chubanshe, 1993.

[CR68] Zhu Zhenheng 朱震亨 1347b Inquiry into the Propensities of Things 《格致余論》. Beijing: Renminweisheng chubanshe, 1993.

[CR69] Zhu Zhenheng 朱震亨 1481 Teachings of [Zhu] Danxi 《丹溪心法》. Beijing: Renminweisheng chubanshe, 1993.

